# COPD-patients’ perspective on adherence to therapy and its integration into a systematic literature review

**DOI:** 10.1186/s12890-026-04102-8

**Published:** 2026-01-13

**Authors:** Maximilian Zimmermann, Doreen Kroppen, Omar Ammous, Daniel S Majorski, Melanie P Berger, Sarah B Stanzel, Johannes F Holle, Falk Schumacher, Tim Matthes, Wolfram Windisch, Maximilian Wollsching-Strobel

**Affiliations:** 1 Cologne Merheim Hospital, Department of Pneumology, Kliniken der Stadt Köln gGmbH, Witten/Herdecke University, Ostmerheimer Strasse 200, Köln, D-51109 Germany; 2 Cologne Merheim Hospital, Department of Neurology, Kliniken der Stadt Köln gGmbH, Köln, Germany; 3 Department of Rheumatology, Krankenhaus Porz am Rhein, Cologne, Germany; 4 Witten/Herdecke University, Witten, Germany; 5 Institute for Medical Statistics, University Medical Center Goettingen, Göttingen, Germany

**Keywords:** COPD, Adherence, Patient involvement, Qualitative research, Systematic review, Patient-centered approach

## Abstract

**Objective:**

Patient involvement in scientific research is becoming increasingly important to ensure a patient-centered approach to medicine. The objective of this study is twofold: firstly, to ascertain patients’ perspectives on adherence to therapy and secondly, to integrate this information into a systematic review (SR) on interventions to improve adherence in chronic obstructive pulmonary disease (COPD).

**Methods:**

In parallel with the SR, two focus group interviews with a COPD self-help group were conducted using semi-structured interview guidelines. The interviews were analyzed using computer-assisted qualitative content analysis according to Kuckartz with the aim of complementing the results of the systematic literature review and to develop propositions for further scientific use.

**Results:**

The first focus group interview comprising 321 codes included 14 (mean age 67.7 ± 6.8 years; 71.4% female) and the second interview comprising 139 codes included 10 (mean age 68,5 ± 8,2 years; 50% female) patients. Ten categories of the content analysis informed the logic models and the applicability analysis of the SR and six propositions representing the patient’s perspective were developed for further scientific use: Main themes were (I) Enhancement of patient’s self-efficacy (II) Access to trusted medical information (III) Patient-physician relationship (IV) Measures to improve adherence (V) Sociocultural/medical environment (VI) Methods to measure adherence.

**Conclusion:**

The inclusion of the patient perspective in an SR can be successfully achieved by conducting focus group interviews. Multimodal measures aimed at enhancing patient’s self-efficacy helping them to achieve individual goals reinforces adherence to therapy from a patient’s perspective.

**Supplementary Information:**

The online version contains supplementary material available at 10.1186/s12890-026-04102-8.

## Introduction

Chronic obstructive pulmonary disease (COPD) is a major health challenge and one of the world’s most prevalent diseases [1]. COPD was directly linked to 3.23 million deaths in 2019, underlining its worldwide importance [2]. Exacerbations are responsible for two-thirds of the economic burden of COPD-related health costs [3–6]. Exacerbations are partly the consequence of low adherence to therapy in COPD patients and result in higher exacerbation frequencies [7]. Each severe exacerbation increases not only the economic burden but also patients’ mortality [8, 9]. During the past century research began to include the patients’ perspective, primarily evident in the frequent use of patient-centered outcomes such as health-related quality of life that is also negatively influenced by exacerbations [4, 10–12].

Therapy adherence is an essential aspect to prevent exacerbations and disease progression in COPD [13–15]. According to the World Health Organization’s (WHO) definition, therapy adherence is defined as the extent to which a person’s behavior – taking medication, following a diet, and/or executing lifestyle changes – corresponds with agreed recommendations from a health care provider [16]. A patient-centered medicine, however, encompasses the understanding that patients are not just passive recipients of care, but active participants in the healthcare system. It is characterized by a confluence of personal, societal, and economic determinants, often contrasting with clinical narratives that predominantly revolve around prescription adherence and regular check-ups.

Over the past decade, several studies have investigated adherence in COPD patients suggesting that it is more complex than adherence in other diseases because it involves not only the quantity of medication taken, but also the quality of the therapy. This becomes evident when one considers the largest pillar of COPD therapy, inhaled therapy. Here, besides the regular intake of the inhalatives at the right time, the quality of the inhalation process and the inhalation technique, is also of utmost importance. Due to multiple factors, Inhaler use is in practice error-prone and in clinical practice often overlooked [17–19]. However, the different perspectives on adherence to therapy and adherence enhancing interventions of healthcare providers and patients might hold potential for antagonistic discussions [20–22].

The present study aims to capture patients’ perspectives on adherence to COPD therapy and enhance intervention strategies. It describes the qualitative process of generating propositions on treatment adherence from two focus group interviews with COPD patients, and the integration of these perspectives into a systematic literature review published elsewhere [23].

## Methods

The study was conducted as a single-center explorative qualitative study at the Department of Pneumology, Cologne-Merheim Hospital, University Witten/Herdecke, Cologne in cooperation with the department of medical statistics of the University Goettingen as part of research project funded by the Federal Ministry of Education and Research in Germany (Bundesministerium für Bildung und Forschung in Deutschland). The study was approved by the Ethics Committee for Human Studies at the University of Goettingen (Application Number: 36/9/22). Data collection was performed in accordance with the ethical standards laid down in the Declaration of Helsinki (last revision: 2013) and written informed consent was obtained from all subjects [24].

### Aim of the study

As a qualitative component of a larger mixed-methods project, the aim of this sub-study was to explore and synthesize COPD patients’ perspectives on barriers and facilitators of treatment adherence through qualitative content analysis of focus group interviews. A further objective was to integrate these patient-reported themes into the interpretation and contextualization of findings from the systematic review, thereby contributing to the development of patient-centered outcome criteria in adherence research.

### Eligibility criteria for participation in the focus group interviews

Participants were eligible if they were members of a COPD support group and had a confirmed diagnosis of COPD. Other inclusion criteria included being 18 years of age or older, having sufficient written and oral German language skills, and being able to give informed consent. To ensure impartiality, individuals with a personal or commercial relationship with members of the research team were excluded. Exclusion criteria included severe cognitive or physical impairments that would prevent active participation in the discussion.

### Analysis of the interview situation, background of the interviewers, preparation of the interview transcripts and purpose of the analyses

Two focus group interviews were conducted to explore the patients’ perspective on adherence to therapy and its influencing factors.

### First focus group interview

The first was conducted after a regular meeting of an independent COPD self-help group on 07.11.2022. During the initial meeting, the interviewers presented their professional background, disclosed any potential conflicts of interest, and provided an overview of therapy adherence to establish a theoretical foundation for the discussion. The interview was conducted using a semi-structured interview guide (Additional file 1). The interviewers (MZ, DK, MWS) were two male physicians specializing in internal medicine and pneumology (MZ, MWS) and one female specialist nurse for intensive care and respiratory therapy (DK). The roles during the interview were divided between moderating the interview, documentation, and handling technology. The interview was conducted as freely as possible to capture the patient’s own concept of adherence to COPD therapy. Its objective was to form a category system representing the patients’ concept of therapy adherence with a focus on motivational and distracting factors.

### Second focus group interview

The second focus group interview was conducted after another meeting of the same COPD self-help group on 06.02.2023. Before the discussion began, the interviewers presented a structured summary of the findings from the systematic literature review on interventions that enhance adherence in COPD patients. They emphasized the types of interventions and outcome measures evaluated in the included studies, as defined in the study protocol published elsewhere [23]. The first part of the interview invited participants to reflect on these findings in relation to their perceived relevance, plausibility and applicability to their personal experiences. The second part of the discussion focused on participants’ experiences of COPD-related interventions, both formal and informal, as well as their views on future measures to promote adherence. Participants were encouraged to articulate patient-relevant outcomes that they would consider meaningful in the context of future studies.

The outline was further used as a semi-structured interview guideline for the second focus group interview (Additional file 1). The roles during the interview remained the same. The objective was to form a category system specifying patients’ perspective on adherence enhancing interventions and clinical study outcome measurements.

### Technical analysis

Both interviews were recorded using a conference microphone and audio recording device and notes were taken to support transcription and to account for special situations. The interview recordings were transcribed and anonymized using transcription software (f4xspeech recognition, dr. dresing & pehl GmbH) and were adjusted and corrected by the coders during initial text analysis using a predefined transcription rule. Both category systems were formed with the objective of deriving propositions from the joint analysis of both category systems.

### Qualitative content analyses

To account for the different aims of the two analyses to different approaches of category formation were used. For the first analysis the inductive category formation via open coding was applied. For the second analysis the deductive-inductive category formation was applied. Both analyses were carried out computer assisted using MaxQDA (Version 2022) and Kuckartz method of qualitative content analysis and quality standards [25]. Moreover, the SRQR and COREQ statements for reporting qualitative research were used as a guideline for presenting the analyses [26]. For the first analysis two of the previous interviewers coded the interview material independently. Consensual coding was used to ensure the quality of the category system. The third interviewer was consulted if differences had to be debated. After the first coding run a conference consisting of the three coders was held to form and discuss the first coding paradigm. In total three coding runs were performed before the final category system was defined. For the analysis of the second focus group interview, a deductive category system was defined by the same three coders who conducted the first analysis, building upon the results from the first analysis and incorporating the interventions and outcome measurements extracted during the systematic literature review that were presented to the patients before the second focus group interview. One coder coded the data with the deductive system and made notes if relevant interview material did not fit the category system. A second coder recoded the data. In a conference the deductive-inductive category system was defined by the two coders, and the material was recoded using the new category system leading to the final category system. Again, consensual coding was used to ensure the quality of the results and a third coder was consulted to resolve open discussions. The selection unit consisted of the transcripts of each interview of the two focus group interviews. Codes were defined as content-bearing text units. The final category systems contain thematic and analytical categories. Themes function as overarching main categories that encompass related categories.

In conclusion, both category systems were analyzed by all coders and the propositions were developed in a consensus conference.

### Integration of qualitative data into the systematic literature review

Qualitative findings from two focus-group interviews with a COPD self-help group were incorporated into a systematic literature review via a two-step synthesis process. The results of the first focus group informed the development of system-based and process-oriented logic models and guided the extraction of categories relating to patient-perceived distractions and motivations. The extracted data on adherence-enhancing interventions and outcome measures then informed the design of the interview guide for the second focus group. The synthesis workflow and protocol are described in detail elsewhere [23].

## Results

### Integration and augmentation of the systematic review

**Fig. 1:**
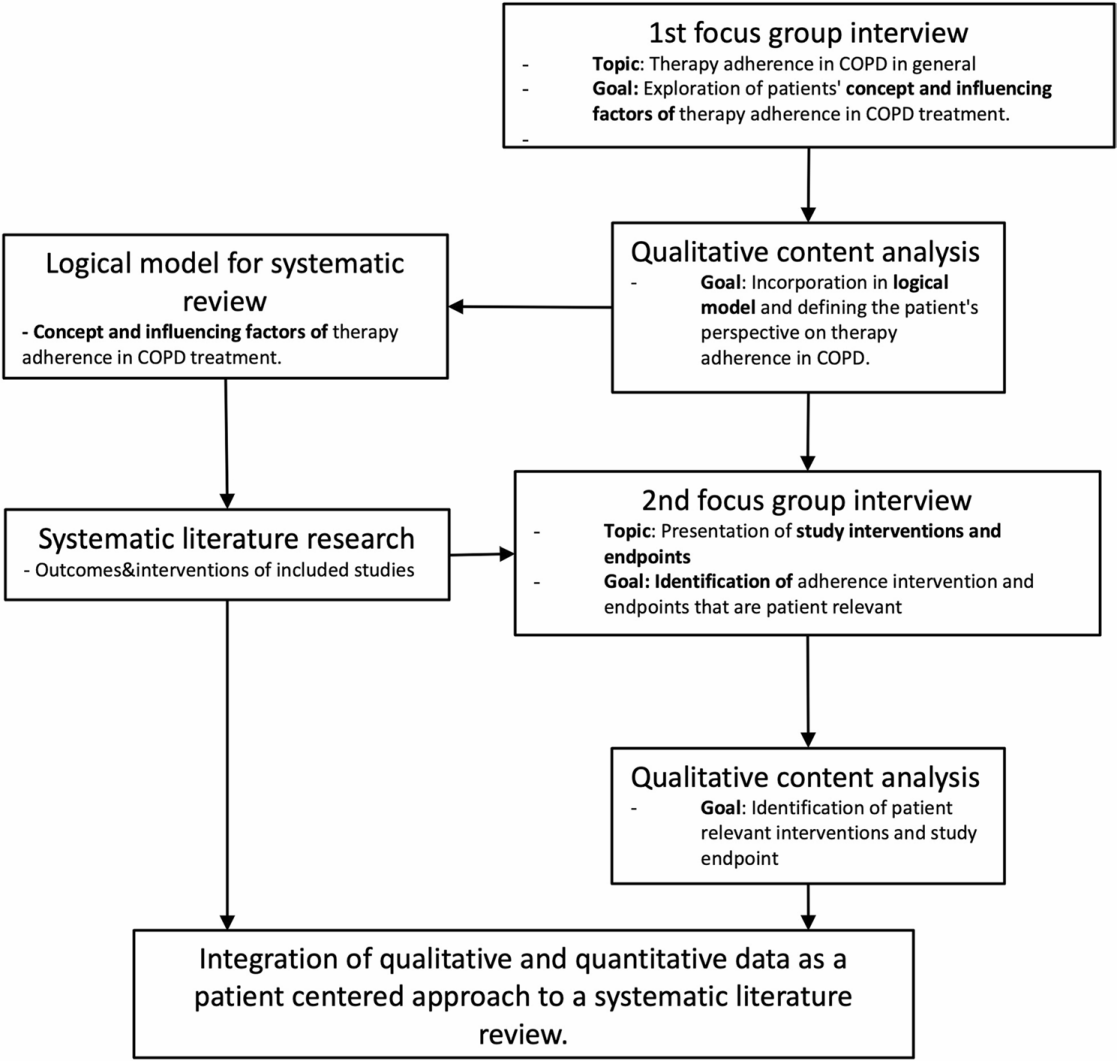
Concept for integrating the patients’ perspective on adherence to therapy in COPD into a systematic literature review

The integration produced two main outcomes. Firstly, the initial focus group generated patient-centered categories (Table 1) that informed the review’s logic models and emphasised patient-perceived distractions and motivations relevant to adherence. Secondly, the second focus group enhanced understanding of patients’ perspectives on interventions and outcome measures that enhance adherence, as used in the included trials (Table 2). This enabled an analysis of applicability from the perspectives of both medical experts and patients. The data-synthesis workflow is summarised in Fig. 1, and the full integrated qualitative analysis has been published elsewhere [27].

**Table 1:** Final category system of the first focus group interview with analysis matrix: Patient-centered factors influencing therapy adherence in COPD

First Focus Group Interview (Self-Help Group COPD); Duration: 01:16:43 h; 14 Patients; 321 Codes			Analysis of the categories:	
Analytic Categories (Number of Codes)			**Distractors** (36)	**Motivators** (31)
			Distractions/Motivators complement and sometimes mutually influence each other. The removal of Distractions/Motivators can therefore have the opposite effect. The most significant aspects of the main categories are listed as keywords. .	
Thematic Categories (Number of Codes)				
C1	Emotion&Reflection (43)		Resignation; Self-abandonment; Hindrance to participation in life; Lack of self-efficacy; Fear of symptoms.	Overcoming “one’s weaker self”; Establishing a routine; Self-efficacy; Controlling symptoms; Quality of life; Participation in life; Fear of symptoms; Knowledge promotes self-reflection.
	1.1	Concept of Illness&Refelction		
	1.2	Quality of Life		
	1.3	Fears		
C2	Interaction&Information (45)		Unreliable internet sources; Insufficient training by medical staff; Information without interactive options; Hard-to-access information.	High-quality understandable information; Various sources; Positive and interactive communication; Trustworthy internet sources.
	2.1	Sources of Informations		
	2.2	Interaction&Communication		
	2.3	Digital Infrastructure		
C3	Physicians Role (51)		Time pressure; Lack of information about side effects and habituation; Non prioritization of quality of life as a treatment goal; Neglect of comorbidities; Lack of clear treatment concept.	Detailed physicians’ consultation at the onset of the illness; longitudinal education; consideration of the individual situation; discussion of different treatment options; trustful relationship.
	3.1	Physician-Patient-Communication		
	3.2	Training by Physicians		
	3.3	Medical Treatment		
C4	Treatment Methods (71)		Side effects of treatment forms; lack of effect on symptoms or quality of life; complicated handling; insufficient availability.	Therapy options that require adherence; quality of life as a treatment goal; leisure activities as a treatment goal; daily routines; reminder calendars; rehabilitation measures; any form of physical therapy; legal regulations.
	4.1	Research&individual therapy forms		
	4.2	Inhalative Therapy&Smoking cessation		
	4.3	In-patient Rehabilitation		
	4.4	Exercise Therapy		
C5	Structures (44)		Shortage of doctors; time pressure; bureaucracy; non-reimbursement by health insurance companies; difficulty accessing to entitled social benefits.	Interactive social contact with other patients (self-help groups, lung sports groups); family support; supportive social benefits.
	5.1	Healthcare System		
	5.2	Sociomedical Aspects		
	5.3	Sociocultural Environment		

**Table 2:** Final category system of the second focus group interview: Patient-centered interventions and outcome measures

Second Focus Group Interview (Self-Help Group COPD); Duration: 1:03:05 h; 10 Patients; 139 Codes		Analysis of the categories:
	Category Name (Number of Codes)	**Keywords**
C6	Therapy Adherence Measures (17)	Step count measurement (exercise), Qualitative control of inhalation process, Quantitative inhalation controls, Digital methods of adherence measurement, No sufficient picture of adherence behavior, Adherence measurement questionnaires, Disease-specific questionnaires, Individual therapy goals, Individual living situation
C7	Diagnostic procedures & objectifiable health status (24)	Pulmonary function parameters and walking distance as core elements, discrepancy between subjective perception of illness and diagnostics, pulse oximeter (controversial), hospitalisation rate and costs
C8	Telemedical Intervention (10)	Video telephony, digital follow-up, combination with face-to-face interaction, non-medical training, auditory and visual reminders for therapies, ease to use, no additional costs, digital literacy
C9	Interventions (44)	Face-to-face training rated most effective, human bonding has positive effect on adherence, Regular repetition, Training by pharmacists, nurses etc. rated positive, Use of known inhaler devices, Counters, time as limiting factor, Multi-modal approaches rated positive (rehabilitation), Group therapy rated as promoting adherence (lung sports)
C10	Subjective Perception of Illness (44)	Dealing with helplessness, fears, doubts, frustration with the health care system, desire for empathy from practitioners, taking mental health into account, social obligation to participate in treatment and training, personal initiative in the disease management process

### Description of the focus group interviews

#### First focus group interview

In the first focus group interview 14 patients participated (age 67.7 ± 6.8 years (mean ± SD); 71.4% female). The interview lasted for 76 min and 6.7 ± 5.5% (mean ± SD) speaking time per patient was recorded. All of them had been treated by a general practitioner and a specialist in pneumology. Patients had been suffering from COPD for 13.1 ± 7.7 years (mean ± SD). 12 reported taking inhalation therapy regularly, eight reported taking COPD medication in tablet form. Seven used long-term oxygen therapy (LTOT), three used some form of non-invasive ventilation. 12 were doing some form of exercise therapy on a regular basis and all regularly attended self-help group meetings.

The first focus group interview qualitative content analysis compiled of 321 codes. The category system contained five main categories. The categories were divided into subcategories for further specification. Themes function as overarching main categories that encompass related categories. Two analytic categories, “Distractors” and “Motivators”, served as influence categories on adherence, classifying adherence-hindering and adherence-promoting attributes within each thematic category in the first category system. The category system and keyword analysis charts are presented in Table 1.

#### Second focus group interview

In the second focus group interview 10 patients participated (age 67.1 ± 8.8 years (mean ± SD); 60.0% female). The interview lasted for 63 min and 10.0 ± 8.0% (mean ± SD) speaking time per patient was recorded. All of them had been treated by both a general practitioner and a specialist in pneumology. On average, the patients had been suffering from COPD for 14.7 ± 7.3 years (mean ± SD). All reported taking inhalation therapy, five reported taking COPD medication in tablet form. Three used long-term oxygen therapy, none used a form of non-invasive ventilation. Nine were doing some form of exercise therapy on a regular basis and all regularly attended self-help group meetings. 57% of COPD patients that participated in the first interview also participated in the second focus group interview.

The second focus group interview qualitative content analysis compiled 139 codes. The category system contained five main categories. Both category system and keyword analysis charts are presented in Table 2.

#### Patient-centered propositions for improving adherence

Following the analysis of the two category systems derived from the qualitative content analysis, six propositions were developed to promote a patient-centered approach to improve therapy adherence in COPD. These propositions are detailed below.

##### Proposition I

Empowering COPD patients’ self-efficacy and addressing disease-related fears, improves their quality of life and reinforces adherence to therapy.

Patients described being motivated by feeling empowered to control their symptoms. Supportive factors include the establishment of routines, knowledge about one’s own condition and the psychological processing of the diagnosis. A sense of self-efficacy was described as a strong reinforcement for adherence, whereas resignation and self-surrender hindered adherence. Improvement in quality of life was mainly articulated through being able to participate in social life. Even in direct comparison with mortality, patients preferred quality of life as study endpoint and suggested including individual personal goals as endpoints. Anxiety about the disease is reported to play an ambivalent role. On the one hand, they promote adherence in the hope to prevent exacerbations of symptoms. On the other hand, they lead to non-adherence in form of resignation.

*FoGo1, Line 164: Moderator: What motivates you then?*

*Patient 6: Life [itself]. That I can still walk around outside*,* that I can tolerate myself. […] Patient2: […]*,* that I can dance with my grandson. At his wedding […]*.

##### Proposition II

Accessible and trusted information from medical experts via analogue and digital resources enable patients to gain knowledge about their disease, which supports self-efficacy and adherence to therapy.

Patients emphasize that adherence can only occur if sufficient accurate information is provided. The main sources of information, according to patients, are physicians, pharmacists, hospitals, rehabilitation centers, and health insurance companies. The internet and apps are mentioned as a frequently used source, but the quality and availability of information is criticized. Information events (e.g. summer festivals of self-help groups) are considered as important for specialized information about the disease and treatment options.

##### Proposition III

Clear communication and a trusting relationship between therapists and patients form the basis of treatment adherence. Specific training in treatment methods is just as important as consideration of the individual situation and therapeutic goals.

A trusting relationship with the physician was highlighted as crucial for adherence to therapy. Moreover, a strong desire for human interaction and empathy was expressed. Clear communication of the diagnosis and individual consideration of the current life situation and co-morbidities were seen as important.

*FoGo1*,* Line 98: Patient4: And that’s when I quit […] after 40 years of smoking. Half a year later*,* I was diagnosed with COPD. […] my very first pulmonologist offered me a COPD and asthma training. […] I haven’t heard that again from any doctor since. Yes*,* and it was really good. […] Everything was explained to you in a human way*,* how to use the device*,* what’s available and so on…. None of the doctors afterwards offered all of that.*

##### Proposition IV

Device-specific training in inhaler technique, therapies with rapid treatment success, multimodal treatment approaches and therapies that include physical exercise are likely to improve adherence. Easy-to-use telemedical interventions (e.g. reminders) can support but not replace personal interaction, while repeated face-to-face visits with multimodal interventions aimed at building routines were rated as highly effective in supporting adherent behavior.

Face-to-face training was considered to be a very efficient intervention. This was particularly emphasized for inhaler training, ideally with scheduled repetitions. While training by physicians was highlighted as one of the most important adherence-enhancing interventions, patients were less concerned about who provided the inhaler device training as long as the person had appropriate medical competence. The use of the devices was generally described as complicated, and medication side effects were also reported to reduce adherence. A well-structured treatment plan, coupled with appropriate education, provides reassurance, patients said.

*FoGo1*,* Line 272: Patient2: What’s also important - the application. Because every spray is different. There are the Easyhalers*,* there’s powder. You must take a deep breath in and out first to learn how to use it*,* and then you must press. Some people push*,* but they’re already breathing out*,* not in. And this is what you learn in these training sessions.*

*Patient1: Inhale and exhale. It’s so complicated when you do it. Yes*,* it really is. Sometimes you don’t even have the air to do it. Yeah.*

*FoGo2*,* Line 89: Moderator: What about the training that is offered? Does it have to be done by the doctor? Or is it okay if it’s done by the nursing staff? Are you basically indifferent?* […]

*Patient9: It must be someone who knows about this - it doesn’t matter if it’s a doctor*,* a nurse*,* a pharmacist*,* or whatever. It must be someone who really knows about it.*

Multimodal therapy approaches in rehabilitation services, including psychological aspects were highlighted as being particularly important in promoting adherence. Therapies involving physical exercise or group sessions such as pulmonary exercise, strength training, gymnastics, breathing exercises, and yoga were mentioned as beneficial. Through physical training and exercise, routines were developed that led to or promoted adherent behavior.

*FoGo1*,* Line 480: Patient9: I was in […] rehabilitation*,* and it was the best thing that could have happened to me. The doctors*,* they were unique […]. They helped me very*,* very much. I started doing sport there and […] I don’t want to be without it anymore*,* because it helps me breathe better.*

##### Proposition V

Meeting like-minded individuals (e.g. COPD self-help groups) and support by family and friends are crucial to overcome barriers to therapy adherence.

Patients had the impression that physicians do not have much time, mainly due to the health care system, and that it is difficult to get an appointment with a specialist, which they described as adherence hindering. Sociomedical aspects play an important role for patients in terms of their disease course and quality of life (e.g. applying for disability benefits, a disability card, and level of care). The socio-cultural environment was consistently described as important in promoting and motivating adherence. The social network of like-minded people in self-help and pulmonary exercise groups were often associated with fun and humor and a positive feeling about therapy. Family support was also described as essential.

*FoGo1*,* Line 36: Patient5: I would like to add how valuable our self‑help group is. To this day I would not have a disability ID card or an assigned care level (i.e.*,* in Germany: a formal assessment that determines eligibility for long‑term care benefits)*,* both of which the group helped me obtain. These are examples of what the group has made possible for me.*

##### Proposition VI

Measurement of adherence should include multiple aspects, including objective (e.g. inhaler dose counting/inhaler technique) and subjective measurements such as accurate disease- and therapy-specific questionnaires. Moreover, regular assessment of objective health status and individual goals supports adherent behavior.

To improve adherence at home, some patients described using different reminder techniques for inhalation, while others reported being reminded by their symptoms. Dosage and step counters were found to be effective. Patients expressed that they were generally receptive to digital approaches, but concrete examples were only superficially discussed. Digital applications that allow for data sharing with healthcare providers were adherence-enhancing. Reminders for inhaled medication were described as useful. Auditory and visual cues were positively highlighted. Telehealth interventions should be as simple as possible and should be accompanied by training. It was important that there were no additional costs.

*FoGo2 134: Moderator: […] Telemonitoring. […] Is this something where you would say*,* in principle*,* it could be helpful*,* or where you would say*,* no*,* not so interesting? […]*

*Patient5: But I would say*,* all in all*,* it’s not a bad idea. There is video telephony. Basically*,* it doesn’t matter if the nurse or the doctor is sitting right in front of me or if he’s talking to me via video.*

It was emphasized that relying solely on objectively measurable adherence methods is not sufficient. Patients emphasized the need for precise disease-specific questions to accurately assess the level of adherence, covering specific aspects of behavior and related symptoms. Patients suggested a multimodal holistic approach for effective adherence measurement. Pulmonary function tests were seen as a key diagnostic tool. The use of diagnostic tools such as pulse oximeters were discussed ambivalently, depending on the situation. Other desired parameters to assess health status included walking distance, hospitalization rate and disease-related costs. The direct linking of interventions to the measurement of disease progression was viewed positively.

#### Joint analysis of the category systems and resulting propositions

**Fig. 2:**
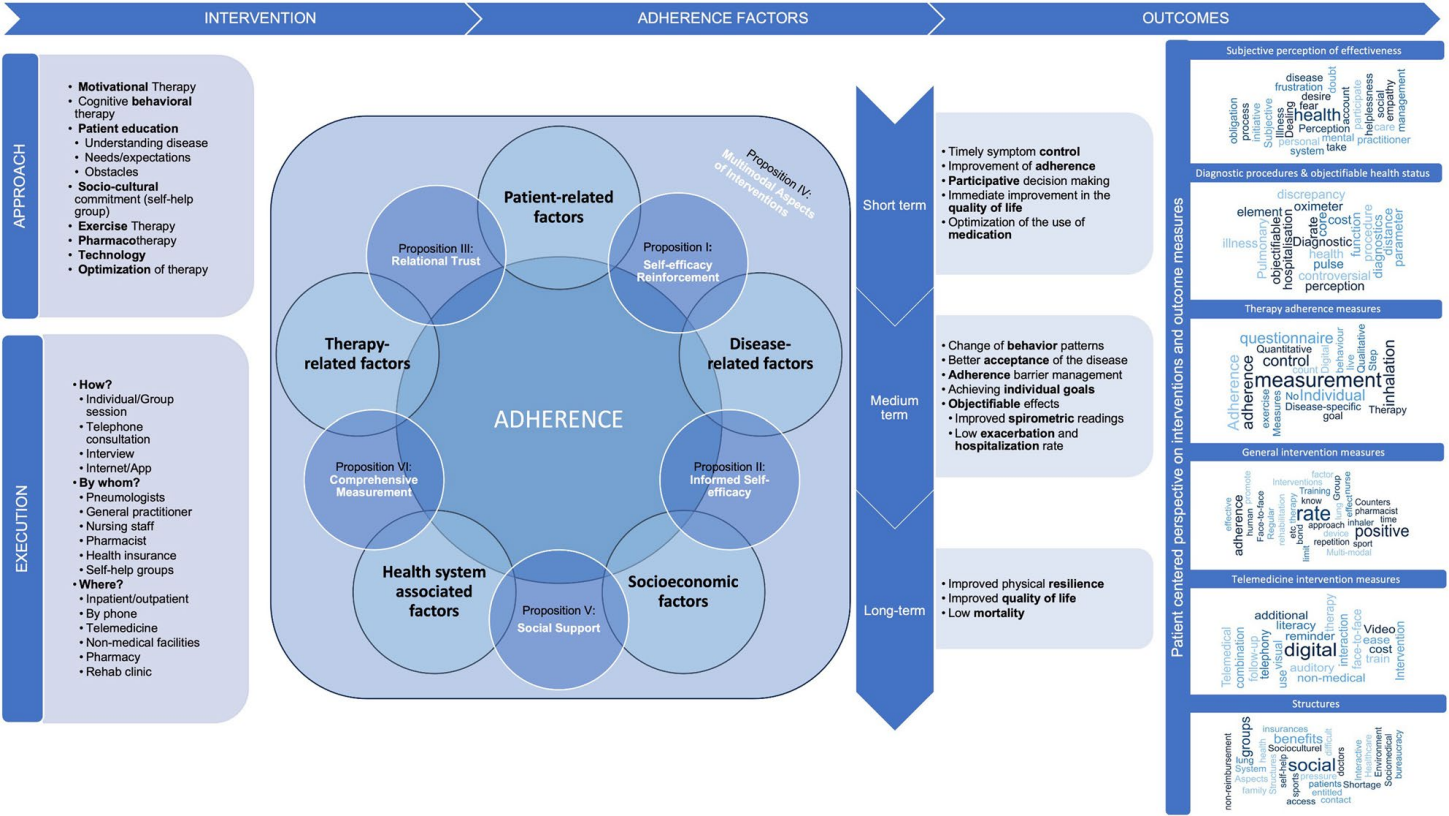
Logical model of the patient’s perspective on the concept of adherence to COPD-therapy including the six propositions Notes: This figure illustrates the integration of category systems from two focus group interviews and the inclusion of six propositions into the current logical model regarding therapy adherence. It provides an comprehensive overview of patients' perspectives on COPD treatment adherence. The left column displays codes related to approach and execution from the qualitative content analysis. The central section highlights the current understanding of adherence, combined with six hypotheses from the qualitative content analysis. These hypotheses are strategically placed within the graphic to connect the various elements of the adherence concept, serving as quality indicators for achieving good adherence. The central column outlines patients' perspectives on essential concepts for achieving adherence, from short-term to long-term. The right-hand column displays word clouds that show the study outcomes and interventions identified as effective for ensuring patient adherence, as determined during the second focus group interview. This arrangement highlights the role of the hypotheses in bridging different aspects of the adherence model and emphasizes their potential as markers of quality in achieving optimal adherence

Joint analysis of the two focus group category systems produced six empirically based propositions for patient-centered measures to improve therapy adherence in patients with COPD, as described in detail above. Figure 2 presents a logical model that integrates these propositions and the category systems, providing a comprehensive overview of patients’ perspectives on therapy adherence.

## Discussion

To the authors’ knowledge this is the first study to describe a comprehensive mixed method approach to a systematic literature review informed by the patients’ perspective using qualitative content analysis. The main results can be summarized as follows: Qualitative content analysis can be used to augment a systematic literature review by informing logical models and the applicability analysis from a patients’ perspective. The patients’ views on adherence to therapy enrich the understanding of adherent and non-adherent behavior and the developed six propositions can be used to apply the patients’ viewpoint to existing and future interventions and to guide further research to assess their applicability. Figure 2 provides a visual representation of how the findings from both focus groups, as detailed in the Results section, integrate to provide a comprehensive overview of patient perspectives on COPD adherence.

Medical research is increasingly focusing on the concept of patient participation [16, 28]. Overall, an increasing number of studies have shown that participatory decision-making in medical practice results in better therapy outcomes [10, 29, 30]. As a result, so-called patient-relevant endpoints must be planned in study designs and patient involvement is increasingly demanded by public funders in project applications [31–33]. In the design of systematic literature reviews, the applicability analysis by medical-clinical experts in the field of the research question is already established [34, 35]. For research questions directly related to patient behavior, such as adherence in this study, it is natural to involve patients, as well as medical experts. In the authors’ opinion, two points in the literature research process are particularly suitable for incorporating patients’ opinions. Firstly, in the creation of logical models in preparation for the literature search and, secondly, in the applicability analysis of the individual included studies themselves. Patients’ opinions make it possible to directly evaluate the study results and the interventions used, as well as relating them to existing concepts on the topic to enable a deeper understanding of mutually influencing factors, which is not possible through statistical analysis alone.

A central superordinate concept when analyzing the category systems was patients’ self-efficacy regarding their illness and symptoms. In both category systems, patients described interventions or factors as particularly effective if they were aimed at empowering them to act to improve their symptoms. This concept has partly been described in other studies looking at therapy adherence from a patients’ perspective [36, 37]. It is particularly interesting when considering a widely accepted patient-centered outcome measure that was also rated as important by the patients in this study, namely quality of life. In this study, quality of life was mainly described as the ability to actively participate in everyday life and special (family) events which most of the time required being physically active. Here, patients even suggested using these individual “goals” as study endpoints in order to measure if a therapeutic intervention was successful. However, a therapist-patient relationship that takes into account the individual’s life situation is needed to define these goals. This was also described as essential by the patients in this study.

Although several studies have reported short-term improvements in adherence, these improvements were neither profound nor sustainable [38–40]. Achieving long-term adherence should therefore be a therapeutic goal especially in chronic disease such as COPD. Some studies have reported various reminder techniques (digital, non-digital) to support adherent behavior [41, 42]. Daily routines, however, should be considered separately. It has been shown that routines can influence not only intentional behavior but also above non-intentional behavior, which has been shown to have a major influence on adherence to therapy [43, 44]. Patients in the present study reported that especially therapies that include physical exercise helped them build healthy routines. Moreover, patients were enabled to achieve personal goals associated with an improvement in quality of life uniting two aspects of adherence enhancement.

The formulated proposition intentionally focuses on adherence enhancing aspects. While distractors for adherence were displayed in the results and analyzed, the proposition were designed to inspire or rate action and interventions rather than listing reasons for non-adherence. Moreover, the authors of this study assume that the design of interventions, considering the proposition, can lead to a reflection of individual distractors and therefore help avoiding them.

Future research should focus on the interaction between adherence to therapy, self-efficacy and quality of life and the successful implementation of adherence enhancing routines. Furthermore, clinical studies should be designed to compare interventions that were rated as more efficient by patients with interventions that were not.

The propositions derived from our qualitative analysis may provide a basis for future quantitative studies to further investigate adherence-related factors in COPD and other chronic diseases. By including the patient perspective, this study aims to ensure that adherence research better reflects real-world experiences, rather than relying solely on investigator-driven frameworks. It also provides evidence to support the design of more targeted and practical adherence interventions in routine care.

These findings not only identify key areas for further research but also provide a basis for refining adherence strategies to better meet the needs of patients, ultimately improving adherence support in clinical practice.

This study has limitations due to its qualitative nature. Even though rigorous methodology was transparently implemented, the results cannot be generalized without context. The interviewed patients were regularly visiting self-help group meetings and might therefore be more active than other patients, which might have influenced their answers. Unintentional non-adherence is a major influence factor. Although focus group interviews may have influenced individual responses for various reasons, they not only stimulated discussion but also facilitated self‑reflection, reciprocal exchange about patient experiences, and empowered participants to articulate and negotiate their medical preferences, making this format particularly well suited to the study’s aims.

## Conclusion

Systematic Reviews should include patients’ perspectives. We have developed an approach for including patient perspectives in a systematic review that is mainly based on integrating the results from qualitative content analysis in the logic model for informing the SR protocol (step 1), and the systematic applicability assessment for informing the results of the evidence synthesis (step 2). Our example from adherence interventions for COPD patients suggests that this approach is feasible and can provide valuable insights, which would not have been recognized through wholly quantitative or statistical analysis.

Interventions that strengthen patients’ self-efficacy and help them to achieve individual goals are perceived positively and likely as effective by patients. The formulated propositions can be used to evaluate the suitability of interventions aimed at enhancing adherence to therapy from patients’ point of view.

## Supplementary Information

Supplementary Material 1.

## Data Availability

Dr. med. Maximilian Zimmermann, zimmermannma@kliniken-koeln.de.

## Abbreviations

COPD: Chronic obstructive pulmonary disease
LTOT: Long-term oxygen therapy
SR: Systematic review
WHO: World Health Organization
