# Supplementary material for: COPD-patients’ perspective on adherence to therapy and its integration into a systematic literature review
Source: BMC Pulm Med. 2026 Jan 13;26:27. doi: 10.1186/s12890-026-04102-8 (PMC12849637; doi:10.1186/s12890-026-04102-8)

**Online Data Supplement**

**COPD-patient’s perspective on adherence to therapy and its integration into a Systematic Literature Review**

^1^Maximilian Zimmermann, ^1^Doreen Kroppen, ^5^Omar Ammous, ^1^Daniel S Majorski, ^1^Melanie P Berger, ^1^Sarah B Stanzel, ^2,4^Johannes F Holle, ^3,4^Falk Schumacher, ^5^Tim Matthes, ^1^Wolfram Windisch, ^1^Maximilian Wollsching-Strobel

^1^Cologne Merheim Hospital, Department of Pneumology, Kliniken der Stadt Köln gGmbH, Witten/Herdecke University, Germany

^2^Cologne Merheim Hospital, Department of Neurology, Kliniken der Stadt Köln gGmbH

^3^Department of Rheumatology, Krankenhaus Porz am Rhein, Cologne

^4^Witten/Herdecke University, Germany

^5^Institute for Medical Statistics, University Medical Center Goettingen, Göttingen, Germany

**Semi-structured interview guide for focus group interview 1 on the topic of therapy adherence in COPD patients**

1. Introductory remarks on the study
2. Signing the consent and data protection declaration
3. Written survey of the demographic data of the focus group and individual characteristics
4. Explanation of the tape recording
5. Definition of the terms therapy adherence and chronic obstructive pulmonary disease (COPD) as well as forms of treatment for COPD

**Definition of COPD therapy:**

COPD therapy includes: inhalers, pulmonary sports or physical activity (rehabilitation measures, physiotherapy, breathing exercises, exercises for at home, etc.), long-term oxygen therapy, non-invasive long-term home ventilation, tablet-form medications such as morphine, laxatives, roflumilast.

**Definition of therapy adherence:**

Adherence describes the extent to which therapeutic measures such as medication, diet, or lifestyle changes comply with the recommendations agreed upon with the therapist.

**Time for questions**

**Interview questions:**

**General open questions:**

What helps you personally to adhere to therapy and what do you find particularly helpful here?

What do you think would help others to apply therapy continuously and correctly?

**Personal questions:**

What forms of therapy do you use yourself in relation to your COPD?

How do you integrate these into your daily routine? Have you established a fixed structure? How do you maintain your structure, e.g., when on vacation?

Do you combine your COPD medication with medication for other conditions?

Do family members and/or partners help you with this at home?

Have you been shown how to use your inhaler?

What role does this self-help group play for you in this regard?

Do you use technical aids to help you remember to use your therapies or to learn and perform them correctly?

Is there an invention you would like to see that would help with therapy adherence?

Do you use the internet in this context?

**Question about the doctor-patient relationship:**

Have you had any experiences in which a doctor or staff member at a clinic or practice, physiotherapist, or other healthcare professional gave you good advice, and what was it?

Do you believe that doctors, nurses, etc. can help with this issue, and if so, how, and if not, why not?

**Questions about studies/patient-relevant endpoints:**

In your opinion, what should be researched in studies on the topic of therapy adherence?

What should be asked in studies on this topic?

What are the relevant points for you when measuring treatment adherence?

How should adherence be measured in studies? Through questionnaires, counters on inhalation devices, i.e., technical aids? Do you have any suggestions of your own?

Do you think there should be rewards for therapy adherence, and if so, what should they be? If not, why not?

**Questions at the end of the interview:**

Do you consider yourself to be adherent to therapy? If so, why, and if not, what do you think would help you develop better adherence?

Why do you think some people are less adherent to therapy?

**Semi-structured interview guide for focus group interview 2 on the topic of therapy adherence in COPD patients**

1. Introductory remarks on the study
2. Signing the consent and data protection declaration
3. Written survey of the demographic data of the focus group and individual characteristics
4. Explanation of the tape recording
5. Presentation of the evaluation of the first focus group interview on the topic of therapy adherence
6. Presentation of the results of the systematic literature review by colleagues at Göttingen University Medical Center, with a focus on intervention measures and study endpoints

**Interview guide:**

The presentation on the following topics will be thematic and discussed immediately afterwards. The moderator will serve as a guide, introducing the topics through the presentation and occasionally intervening in the open discussion to bring the focus back to the topic at hand.

**Presentation of the category system:**

The qualitative content analysis of the first focus group interview resulted in a category system with five main categories: emotions and reflection, interaction and information, the role of the physician, forms of therapy, and structure. These main categories were each divided into subcategories and were each related to the topic of therapy adherence. In addition, distractors and motivators were identified for each category.

**Presentation of disease management programs:**

Disease management programs (DMPs) will be presented comprehensively within the framework of the studies conducted in the literature. The focus will be on the implementation of DMPs by various professional groups such as nurses, pharmacists, and physicians.

Another key issue is whether face-to-face consultations and telephone support are equivalent, whether a DMP is sufficient during hospitalization alone or whether it should be continued beyond the hospital stay, and whether the doctor's office is a suitable location for this.

**Intervention measures:**

Measures to increase therapy adherence based on the evaluation of current clinical studies will be presented in detail. Interventions such as motivational interviews, health coaching, robots, and telemedicine measures will be discussed. In particular, the question of whether telephone support is equivalent to video telephony or communication via a robot will be discussed.

**Study endpoints:**

Study endpoints for achieving good therapy adherence will be discussed based on the current study situation. Endpoints measured by questionnaires, scales, or scores will be discussed. Topic-specific endpoints such as satisfaction, self-efficacy, anxiety, depression, nutritional status, and cognitive testing will be put up for discussion.

Objective study endpoints such as exacerbation rate, hospital days, costs, number of inhaler doses used, medication use, duration of NIV therapy, and spirometry, etc. will also be discussed.

**Questions about study/patient-relevant endpoints:**

- In your opinion, what should be researched in studies on the topic of therapy adherence?
- Do you consider the endpoints presented to be meaningful?
- What other relevant points do you think should be used to measure treatment adherence?
- How should adherence be measured in studies? Through questionnaires, counters on inhalation devices, i.e., technical aids? Do you have any other suggestions?
- Do the endpoints presented cover the needs of patients and are they tailored to those needs?

**Questions at the end of the interview:**

- How do you deal with the idea that objectively measurable values differ greatly from your subjective perception?

**Time for questions**

|  | **Standards for Reporting Qualitative Research (SRQR)*** |  |
| --- | --- | --- |
|  | <http://www.equator-network.org/reporting-guidelines/srqr/> |  |
|  | **COPD-patient’s perspective on adherence to therapy and its integration into a Systematic Literature Review** |  |
| **Title and abstract** | | **Page** |
|  | **Title** - Concise description of the nature and topic of the study Identifying the study as qualitative or indicating the approach (e.g., ethnography, grounded theory) or data collection methods (e.g., interview, focus group) is recommended | Page 1 |
|  | **Abstract** - Summary of key elements of the study using the abstract format of the intended publication; typically includes background, purpose, methods, results, and conclusions | Page 2 |
|  |  |  |
| **Introduction** | |  |
|  | **Problem formulation** - Description and significance of the problem/phenomenon studied; review of relevant theory and empirical work; problem statement | Page 3 |
|  | **Purpose or research questio**n - Purpose of the study and specific objectives or questions | Page 3 |
|  |  |  |
| **Methods** | |  |
|  | **Qualitative approach and research paradigm** - Qualitative approach (e.g., ethnography, grounded theory, case study, phenomenology, narrative research) and guiding theory if appropriate; identifying the research paradigm (e.g., postpositivist, constructivist/ interpretivist) is also recommended; rationale** | Page 4 |
|  | **Researcher characteristics and reflexivity** - Researchers’ characteristics that may influence the research, including personal attributes, qualifications/experience, relationship with participants, assumptions, and/or presuppositions; potential or actual interaction between researchers’ characteristics and the research questions, approach, methods, results, and/or transferability | Page 4 |
|  | **Context** - Setting/site and salient contextual factors; rationale** | Page 4 |
|  | **Sampling strategy** - How and why research participants, documents, or events were selected; criteria for deciding when no further sampling was necessary (e.g., sampling saturation); rationale** | Page 5 |
|  | **Ethical issues pertaining to human subjects** - Documentation of approval by an appropriate ethics review board and participant consent, or explanation for lack thereof; other confidentiality and data security issues | Page 4 |
|  | **Data collection methods** - Types of data collected; details of data collection procedures including (as appropriate) start and stop dates of data collection and analysis, iterative process, triangulation of sources/methods, and modification of procedures in response to evolving study findings; rationale** | Page 4 |
|  | **Data collection instruments and technologies** - Description of instruments (e.g., interview guides, questionnaires) and devices (e.g., audio recorders) used for data collection; if/how the instrument(s) changed over the course of the study | Page 4-6 |
|  | **Units of study** - Number and relevant characteristics of participants, documents, or events included in the study; level of participation (could be reported in results) | Page 6 + 7 |
|  | **Data processing** - Methods for processing data prior to and during analysis, including transcription, data entry, data management and security, verification of data integrity, data coding, and anonymization/de-identification of excerpts | Page 5 + 6 |
|  | **Data analysis** - Process by which inferences, themes, etc., were identified and developed, including the researchers involved in data analysis; usually references a specific paradigm or approach; rationale** | Page 5 +6 |
|  | **Techniques to enhance trustworthiness** - Techniques to enhance trustworthiness and credibility of data analysis (e.g., member checking, audit trail, triangulation); rationale** | Page 5 + 6 |
|  |  |  |
| **Results/findings** | |  |
|  | **Synthesis and interpretation** - Main findings (e.g., interpretations, inferences, and themes); might include development of a theory or model, or integration with prior research or theory | Page 6-10 |
|  | **Links to empirical data** - Evidence (e.g., quotes, field notes, text excerpts, photographs) to substantiate analytic findings | Page 7-10 |
|  |  |  |
| **Discussion** | |  |
|  | **Integration with prior work, implications, transferability, and contribution(s) to the field -** Short summary of main findings; explanation of how findings and conclusions connect to, support, elaborate on, or challenge conclusions of earlier scholarship; discussion of scope of application/generalizability; identification of unique contribution(s) to scholarship in a discipline or field | Page 11 |
|  | **Limitations** - Trustworthiness and limitations of findings | Page 12 |
|  |  |  |
| **Other** | |  |
|  | **Conflicts of interest** - Potential sources of influence or perceived influence on study conduct and conclusions; how these were managed | Appendix |
|  | **Funding** - Sources of funding and other support; role of funders in data collection, interpretation, and reporting | Page 4 |
|  |  |  |
|  | *The authors created the SRQR by searching the literature to identify guidelines, reporting standards, and critical appraisal criteria for qualitative research; reviewing the reference lists of retrieved sources; and contacting experts to gain feedback. The SRQR aims to improve the transparency of all aspects of qualitative research by providing clear standards for reporting qualitative research. |  |
|  |  |  |
|  | **The rationale should briefly discuss the justification for choosing that theory, approach, method, or technique rather than other options available, the assumptions and limitations implicit in those choices, and how those choices influence study conclusions and transferability. As appropriate, the rationale for several items might be discussed together. |  |
|  |  |  |
|  | **Reference:** |  |
|  | O'Brien BC, Harris IB, Beckman TJ, Reed DA, Cook DA. **Standards for reporting qualitative research: a synthesis of recommendations.** *Academic Medicine*, Vol. 89, No. 9 / Sept 2014  DOI: 10.1097/ACM.0000000000000388 |  |
|  |  |  |
|  |  |  |


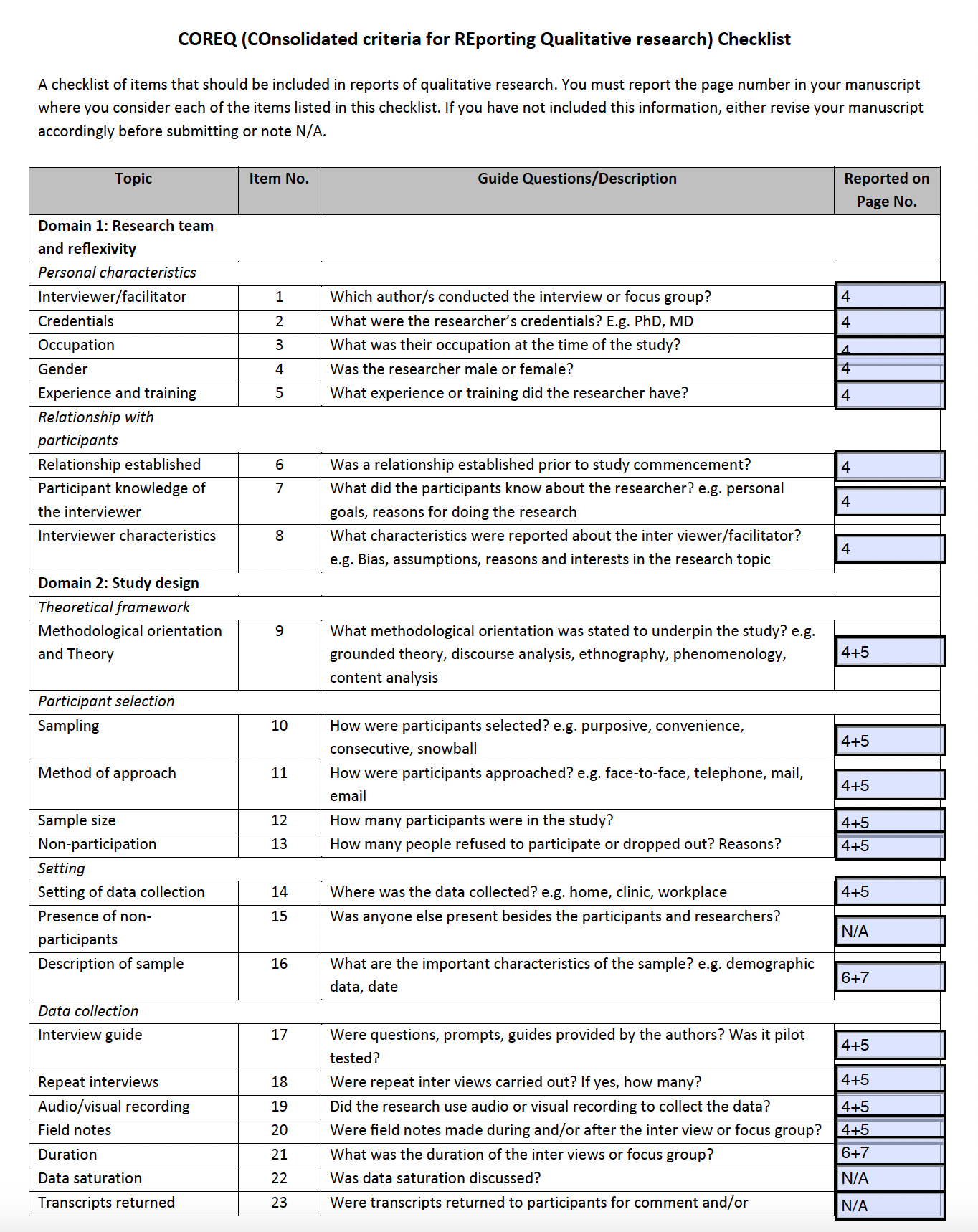


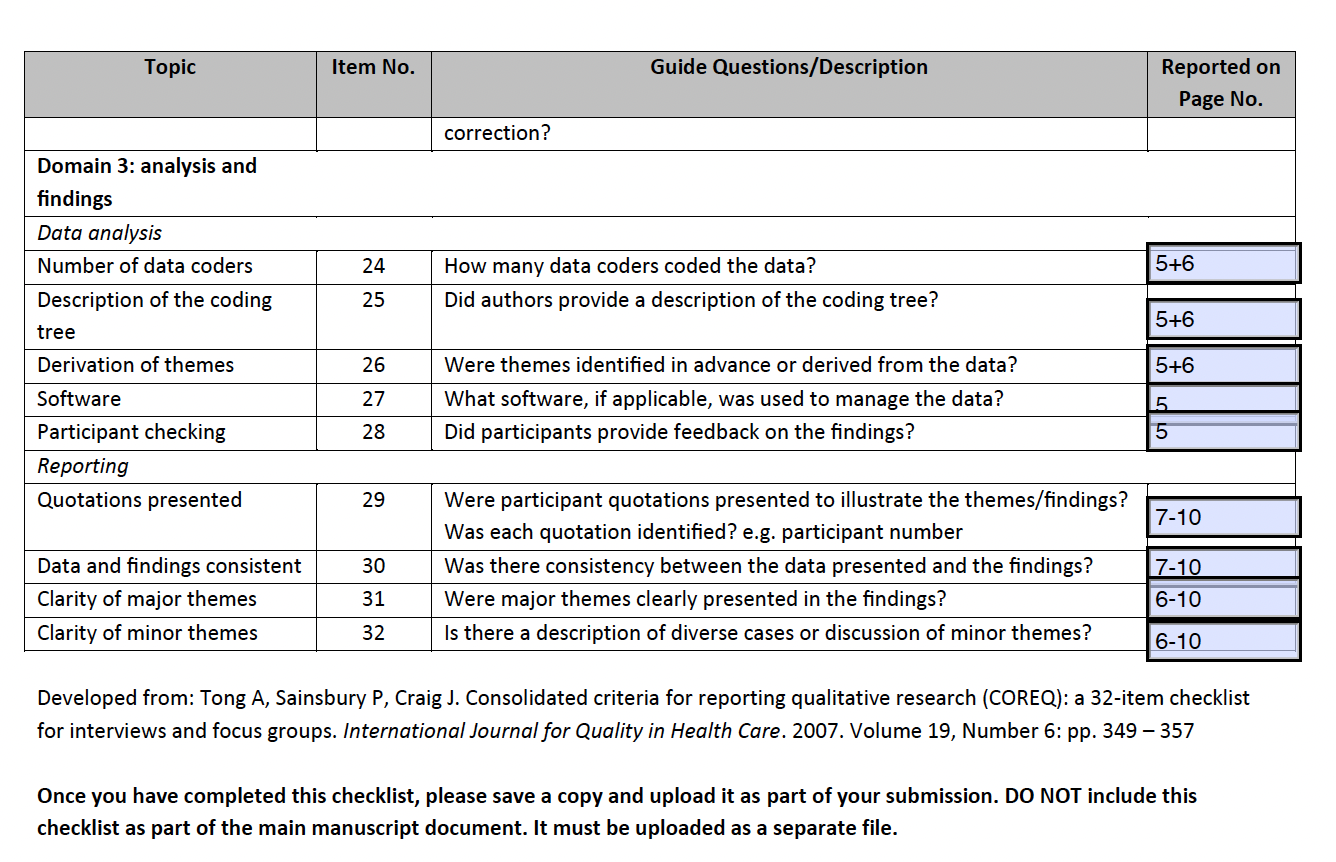

Supplement: Supplementary file 1 — Supplementary Material 1. [file 12890_2026_4102_MOESM1_ESM.docx]
